# Special Issue “The Role of Vitamin D in Human Health and Diseases 4.0”

**DOI:** 10.3390/ijms27031381

**Published:** 2026-01-30

**Authors:** Francesca Silvagno, Loredana Bergandi

**Affiliations:** Department of Oncology, University of Torino, Via Santena 5 Bis, 10126 Torino, Italy

This Special Issue focuses on advancing our knowledge about the role vitamin D plays in human health and disease. The findings from the twenty published papers (nine experimental studies and eleven reviews) provide a consistent and convincing framework demonstrating that vitamin D plays a central role in tissue functions and that impaired synthesis or activity of the hormone or its receptor has inevitable biological consequences. This Special Issue delivers an updated and valuable insight into the complex actions of vitamin D3 and its active form, calcitriol.

A relevant portion of this compendium explores new pharmacologic paradigms. Firstly, considerable effort has been invested in identifying alternative compounds, prompted by the limitations associated with in vivo calcitriol therapy, including side effects such as hypercalcemia and the resistance arising in several pathologies, including cancer. A study by Domzalski and colleagues investigated the antiproliferative and antimetastatic efficacy of natural derivatives of vitamin D3 (VD3) and lumisterol (L3), namely 1,24,25(OH)3D3 and (25R)-27(OH)L3, in different melanoma cell lines. Moreover, a second study synthesized 21 aromatic A-ring 23-oxavitamin D3 derivatives and explored their enhanced selectivity and potency as Hedgehog (Hh) inhibitors, demonstrating that the most promising anticancer activity was mediated by Smo targeting. An alternative approach to the synthesis of new derivatives is enhancing the activity of vitamin D in combination with other molecules, as described by Bergandi and colleagues. In their work, the authors revealed the powerful synergistic anti-inflammatory effect of calcitriol and sulforaphane (SF) in a model of inflamed human retinal epithelium and provided evidence that could be exploited to advance the treatment of age-related macular degeneration. In addition, emerging pharmacologic approaches emphasize personalized supplementation, accounting for interindividual and pathology-related variability in responsiveness to vitamin D and its metabolites. This Special Issue includes a very interesting paper exploring this topic, focusing on a cohort of chronic kidney disease (CKD) patients affected by VD insufficiency, to develop a population pharmacokinetic model of oral cholecalciferol (VD3) and its three major metabolites, 25-hydroxyvitamin D3 (25D3), 1,25-dihydroxyvitamin D3 (1,25D3), and 24,25-dihydroxyvitamin D3 (24,25D3). The reported findings could potentially contribute to the development of personalized dosage regimens for vitamin D treatment in patients with CKD, for whom optimal replacement and maintenance dosing strategies are critical. CKD and the associated vitamin D insufficiency are also the focus of another study included in this collection, in which vitamin D supplementation was found to have significant effects both on the immune system of patients and on in vitro models of vascular tissue and immunity cells. The need for personalized supplementation is further supported by the conclusions drawn by Kalnina and colleagues, who demonstrated that polymorphisms of the GC gene may influence the Latvian population’s susceptibility to multiple sclerosis (MS) and vitamin D status. Genetic variations in the GC gene were associated with significant differences in the serum levels and binding affinity of the encoded vitamin D-binding protein (VDBP), and also individual participants’ vitamin D status. Collectively, these findings underscore the importance of genotype-specific therapeutic strategies for MS management and prevention.

The consequences of vitamin D deficit with regard to diseases were investigated in three experimental works which integrated the results of defects in hormone levels, metabolism, and receptor activity. Notably, all the studies revealed a clear link between defective vitamin D signaling and inflammation or metabolic perturbation. In an experiment focused on alopecia and skin abnormalities, VDR knockout rats exhibited signs of chronic skin inflammation and increased proliferation of epidermal keratinocytes; moreover, the researchers demonstrated that non-liganded VDR was significantly involved in regulating keratinocyte differentiation, proliferation, and cell death in hair follicles and the epidermis. A second study established that vitamin D deficiency (VDD) disrupted metabolic regulation in weaning rats, since perinatal VDD transiently enhanced brown adipose tissue (BAT) thermogenic activity during the animals’ early life. Given that BAT over-activation has deleterious effects on catabolic conditions, the authors suggest that achieving adequate vitamin D levels may represent a target for managing wasting in patients with cachexia. Additionally, not only does vitamin D deficit have a significant impact on tissue metabolism, but conversely, metabolic perturbation can influence the synthesis of calcitriol. A published study on allogeneic hematopoietic stem cell transplantation (HSCT) discovered that kidney function positively correlated with high peri-transplant calcitriol levels and was associated with increased one-year survival following HSCT, although it was not the sole determinant for achieving the protective levels of vitamin D.

Similarly to the experimental studies, some of the eleven reviews addressed vitamin D deficiency (in autism) and supplementation, including its potential role in treating coronary artery disease, autoimmune thyroiditis, and diabetic foot ulcers; the updated information presented in these reviews primarily focuses on inflammatory and immune-based diseases (autoimmune thyroiditis, multiple sclerosis, type 1 diabetes mellitus, inflammatory dermatological disorders, gut inflammation, and lung cancer). Furthermore, many researchers have studied metabolic pathologies (type 2 diabetes mellitus and African Americans subjects with Type II diabetes).

In conclusion, this collection showcases a range of research papers on this crucial topic and their various research directions. These include 1. the discovery of new molecules (derivatives or analogues of vitamin D) and novel combinations which, together with pharmacodynamic studies, can lead to innovative and personalized treatment protocols; 2. investigating vitamin D’s impact on disease, with a special focus on the inflammatory bases of several pathologies.

